# Anti-obesity medications in children and adolescents: blood pressure and chronic kidney disease implications

**DOI:** 10.1007/s00467-026-07293-8

**Published:** 2026-04-25

**Authors:** Margaret A. Clement, Joyce P. Samuel

**Affiliations:** Department of Pediatrics, McGovern Medical School at UTHealth Houston, Houston, TX USA

**Keywords:** Pediatric obesity, Pharmacotherapy, Hypertension, Chronic kidney disease

## Abstract

Pediatric obesity is a growing public health crisis with profound cardiometabolic and kidney consequences extending into adulthood. Emerging frameworks now distinguish preclinical obesity from clinical obesity, emphasizing excess adiposity-related organ dysfunction or functional limitations in activities of daily living. Obesity is a central driver of cardiovascular–kidney–metabolic syndrome, underscoring the need for integrated treatment approaches. This narrative review summarizes current evidence for lifestyle interventions and pharmacologic therapies for pediatric obesity, with a focus on implications for hypertension and chronic kidney disease. Intensive health behavior and lifestyle treatment remains first-line therapy, but access and sustainability barriers limit effectiveness. Pharmacotherapy is increasingly used as adjunctive treatment, with five medications currently approved for non-syndromic obesity in children. Among available agents, glucagon-like peptide-1 receptor agonists (GLP-1 RAs) and combination phentermine/topiramate demonstrate the greatest efficacy in adolescents, while older agents and off-label therapies offer modest benefit with greater limitations. Emerging data suggest GLP-1 RAs may improve blood pressure and kidney outcomes, although pediatric evidence remains limited. Lack of long-term safety data, durability of weight loss, cost, and access remain substantial barriers in the use of obesity pharmacotherapy for children. Sustained, coordinated care across the lifespan is essential to effectively manage pediatric obesity and its cardiometabolic and kidney sequelae.

**Graphical Abstract:**

**Figure:**
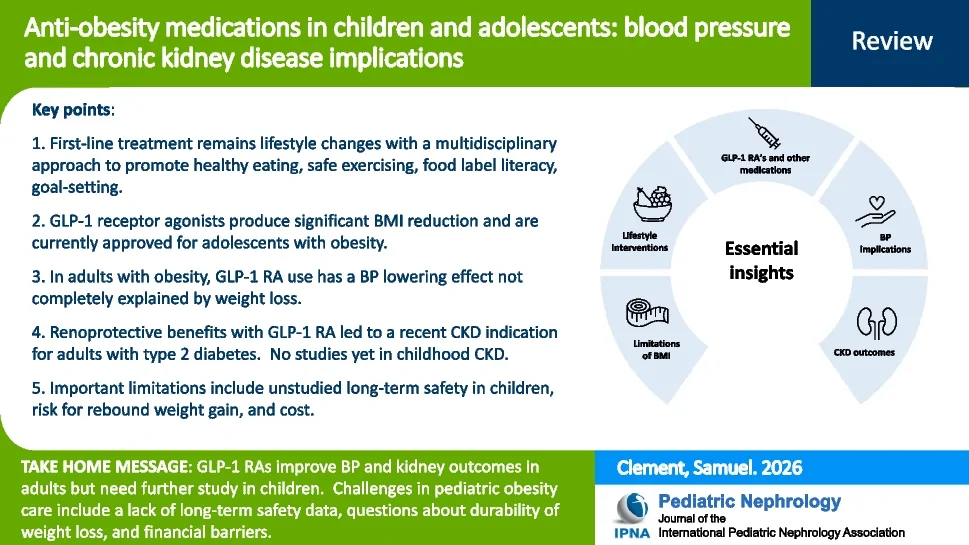
A higher resolution version of the Graphical abstract is available as Supplementary information

**Supplementary Information:**

The online version contains supplementary material available at https://doi.org/10.1007/s00467-026-07293-8.

## Introduction

Pediatric obesity is a growing global public health crisis associated with serious short and long-term health consequences. Definitions of obesity vary across the lifespan, with adult categories based on absolute body mass index (BMI) cut-offs [1], and childhood categories based on either BMI percentiles from length-, age- and sex-matched normative data for children ages 2 to 20 years old [2], or sex-specific weight-for-length charts in children younger than 2 years [3, 4]. “Extended” BMI categories have been proposed to differentiate extremely high BMI values in children, expressed as the percentage above the 95th percentile (Table 1).

**Table 1:** Comparison of current definitions of obesity for children and adults

**CDC Child and Teen BMI categories (age 2–20)** [2]	
Classification	Percentile Range (BMI percentile)
Underweight	Less than 5th %
Healthy weight	5th to < 85th %
At risk for obesity	85th to < 95th %
Obesity	95th % or greater
**American Academy of Pediatrics expanded definition of severe obesity** [14]	
Class 1 obesity	95th % to 120% of the 95th %
Class 2 obesity	120% to < 140% of the 95th % (or absolute BMI 35 to < 40 kg/m^2^)
Class 3 obesity	≥ 140% of the 95th % (or absolute BMI ≥ 40 kg/m^2^)
**CDC Adult BMI categories** [1]	
Classification	Absolute BMI (kg/m^2^)
Underweight	< 18.5
Healthy weight	18.5–24.9
Overweight	25.0–29.9
Obesity	≥ 30.0
Class I obesity	30–34.9
Class II obesity	35–39.9
Class III (severe) obesity	≥ 40

As a standalone screening and diagnostic tool for obesity and its complications, BMI has several well-described limitations. BMI alone cannot distinguish between excess adiposity versus lean body mass and does not differentiate between visceral versus subcutaneous adipose tissue distribution, distinctions which carry important health consequences [5, 6]. In light of these limitations, in 2025 *The Lancet* Commission and the European Association for the Study of Obesity proposed a new framework for the diagnosis of obesity that incorporates other measurements of body size (waist circumference, waist-to-hip ratio, or waist-to-height ratio) in addition to BMI [7]. The updated definition differentiates “clinical obesity”, a chronic systemic disease state directly caused by excess adiposity, from “preclinical obesity”, a condition of excess adiposity without evidence of adiposity-related organ dysfunction or functional limitations in activities of daily living but with increased future health risk.

The prevalence of obesity in children has steadily risen in the last several decades, with current estimates suggesting that nearly 20% of children and teens in the U.S. are affected [8], with disproportionately higher rates noted among adolescents, certain ethnic/racial groups (non-Hispanic Black and Mexican American), and households with lower socioeconomic status [9]. These disparities highlight the environmental, structural, and systemic factors contributing to the development of childhood obesity. Unequal access to healthy foods, safe environments for physical activity, and healthcare resources, combined with targeted marketing of unhealthy foods may contribute to this growing health disparity in the U.S. [10]. The global burden of childhood obesity has tripled in the last 30 years, with the highest rates observed in north Africa and the Middle East (United Arab Emirates and Kuwait) [11].

Cardiovascular–kidney–metabolic (CKM) syndrome, a term first coined by the American Heart Association in 2023, highlights the interconnected pathophysiology of kidney disease, diabetes, heart disease, and obesity [12]. This syndrome identifies excess or dysfunctional adipose tissue as the central driver of a multisystem disease, stressing the need for treating these conditions collectively rather than independently. As anti-obesity medications are increasingly studied and prescribed to children with obesity, a greater understanding of these medications and their impact on kidney health will be particularly relevant for pediatric and adult nephrologists. This review will provide an overview of the current state of anti-obesity treatments in children, including lifestyle interventions and pharmacotherapy.

## Behavioral and lifestyle interventions

Behavioral and lifestyle modifications remain the first-line treatment for obesity in children ages six years and older [13, 14]. The 2024 US Preventive Services Task Force (USPSTF) Recommendation Statement on Interventions for High Body Mass Index in Children and Adolescents concluded with moderate certainty that intensive health behavior and lifestyle treatment (IHBLT) yields a moderate net benefit and should be provided for children and adolescents 6 years and older with obesity (Level B recommendation). The evidence to support the use of IHBLT suggests a strong dose–response relationship, requiring at least 26 contact-hours of family-based treatment over 2 to 12 months [14]. A comprehensive approach involving education and behavioral strategies for healthy eating, safe exercising, caloric balance, food-label literacy, and goal setting is typically delivered by a multidisciplinary team made up of pediatricians, dieticians, psychologists, exercise physiologists, and social workers. Long-term data suggests that behavioral treatments can produce significant and lasting benefits for children with obesity. A randomized controlled trial demonstrated that improvement in BMI and insulin sensitivity were sustained up to two years after intervention, highlighting the long-term benefit of behavioral programs even after active intervention sessions have ceased [15].

Despite their proven effectiveness, IHBLT programs may not be accessible to patients due to individual and systemic barriers [16]. These programs can be time consuming, costly to implement, and require long-term active engagement by the patient, family, and healthcare providers. The effectiveness of IHBLT programs can be limited by families’ lack of engagement, high attrition rates, and the frequency of visits combined with the lack of appointment availability outside of usual work and school hours. When IHBLT programs are not available, pediatricians should utilize motivational interviewing to encourage specific behavior strategies, while collaborating with local specialists and community programs to provide the best locally available intensive treatment for their patients with overweight and obesity.

## Pharmacotherapy

The use of anti-obesity medications among adults has experienced a widespread explosion in popularity in the last five years with the development of highly efficacious medications. In pediatrics, pharmacological treatment is usually reserved as adjunctive therapy for patients who have not achieved sufficient weight loss using first-line behavioral and lifestyle interventions alone [17]. The 2023 American Academy of Pediatrics (AAP) Clinical Practice Guidelines emphasize proactive treatment over “watchful waiting”, and recommend that pharmacotherapy for weight loss *may be offered* to children between 8 and 11 years old diagnosed with obesity and obesity-related comorbidities, and *should be offered* to adolescents ≥ 12 years with obesity alone as an adjunct to health behavior and lifestyle treatment (italics added for emphasis) [14]. The AAP guidelines were met with criticism by some who suggested that the evidence base for obesity pharmacotherapy in children is not yet strong enough to support early and aggressive use of medications in children with obesity [18].

A limited number of newer anti-obesity medications have been studied in children, but most of the trials reported only short-term outcomes, typically with less than one year follow-up. In 2024 the USPSTF concluded that the current pool of evidence was insufficient to fully consider the balance of benefits and harms of pharmacologic treatment of obesity in children, citing the limited number of trials per pharmacologic agent and short duration of follow-up [13]. Furthermore, most of these medications are associated with rebound weight gain when treatment is discontinued, suggesting that treatment might need to be chronic or even lifelong [19]. The following is a review of available and emerging pharmacological agents in use or under current investigation for the treatment of obesity in pediatrics, summarizing trial data on weight loss mechanism, efficacy, safety, and regulatory approval status in pediatric populations.

Five anti-obesity medications have been FDA approved specifically for the treatment of non-genetic obesity in children and adolescents. Approved medications include phentermine (approved in 1959 for short-term use in adolescents ≥ 16 years old), orlistat (approved in 2003 for adolescents ≥ 12 years old), liraglutide (approved in 2020 in adolescents ≥ 12 years old), combination phentermine/topiramate (approved in 2022 in adolescents ≥ 12 years old), and most recently, semaglutide (approved in 2023 for adolescents ≥ 12 years old) [20]. Setmelanotide, an injectable melanocortin 4 receptor agonist, was recently approved for children with certain syndromic or monogenic forms of obesity, but is outside the scope of this review [21]. Given the limited number of approved pharmacologic options, off-label use of other drugs occurs in clinical practice, including metformin, combination naltrexone/bupropion, and topiramate monotherapy. Continued evaluation of long-term outcomes, safety, and relative efficacy will be necessary as pharmacologic therapy assumes a larger role in the treatment of obesity in children (Table 2).

**Table 2:** Obesity pharmacotherapy in children

Drug name and class	Mechanism for weight loss	Route of administration	Pediatric dosing for weight loss	Pediatric indication
**Approved for weight loss in children** (listed in chronological order of FDA approval)				
Phentermine monotherapy; amphetamine analog	Appetite suppression	Oral	Initial dose 7.5–15 mg/day, may titrate up to a maximum of 37.5 mg	Approved for patients > 16 years old with an initial BMI ≥ 30 kg/m^2^, or ≥ 27 kg/m^2^ in the presence of other risk factors
Orlistat; gastrointestinal lipase inhibitor	Decreased fat absorption	Oral	120 mg three times a day with meals	Approved for patients ≥ 12 years old with obesity
Liraglutide; GLP-1 RA	Appetite suppression, increased satiety, delayed gastric emptying	Subcutaneous injection	Initial dose 0.6 mg once daily, escalate by 0.6 mg weekly up to a maximum of 3 mg (maintenance)	Approved for patients ≥ 12 years old with obesity
Phentermine + topiramate; amphetamine analog and carbonic anhydrase inhibitor	Appetite suppression, increased satiety	Oral	3.75 mg/23 mg once daily in morning for 2 weeks, then increase as tolerated to maximum dose 15 mg/92 mg	Approved for patients ≥ 12 years old with obesity
Semaglutide; GLP-1RA	Appetite suppression, increased satiety, delayed gastric emptying	Subcutaneous injection	Initial dose 0.25 mg weekly, escalate by 0.25 mg every 4 weeks up to a maximum of 2.5 mg (maintenance)	Approved for patients ≥ 12 years old with obesity
**Off-label weight loss therapies **(listed in alphabetical order)				
Metformin; biguanide antihyperglycemic	Appetite suppression	Oral	Dosing for T2DM: 500 mg once or twice a day with gradual increase over 1–2 weeks up to max dose of 2000 mg total daily	Approved for patients ≥ 10 years old with type 2 diabetes mellitus Not approved for weight loss in pediatrics or adults
Naltrexone + bupropion; opioid antagonist and norepinephrine/dopamine reuptake inhibitor	Appetite suppression with inhibition of negative feedback loop	Oral	Adult dosing: Initial dose 8 mg/90 mg once daily, escalate dose weekly up to 16 mg naltrexone/180 mg bupropion twice daily (maintenance)	Not approved for any indication in pediatrics Approved for weight loss in adults with obesity or overweight with ≥ 1 weight-related comorbid condition
Topiramate monotherapy; carbonic anhydrase inhibitor	Appetite suppression, reduced binge eating behaviors	Oral	Reported dosing for weight loss in adolescents [88]: 25 mg once daily at bedtime, increase by 25 mg every 2–4 weeks to max 200 mg/day split into twice daily dosing	Approved for patients ≥ 2 years old with epilepsy and ≥ 12 years old for migraine prophylaxis Not approved for weight loss in pediatrics or adults

### Approved weight loss pharmacotherapy

In this section, FDA approved weight loss medications are reviewed in reverse chronological order of FDA approval.

#### Glucagon-like peptide-1 receptor agonists

As the scientific community gains a deeper mechanistic insight into the role of the gut–brain axis in appetite regulation, newer anti-obesity drugs have been developed to directly target these pathways. Naturally occurring hormones, including glucagon-like peptide-1 (GLP-1), glucose-dependent insulinotropic polypeptide (GIP), glucagon, and amylin, are stimulated by nutrient intake and produce widespread effects across organ systems. The activation of GLP-1 receptors has an incretin effect, amplifying insulin release with enteral intake of glucose, along with other pleiotropic effects, including inhibition of glucagon release, delayed gastric emptying, and central appetite suppression. Endogenous human GLP-1 degrades in minutes, but in the early 1990s researchers isolated a structurally similar peptide from Gila monster venom, exendin-4, with a longer circulating half-life [22]. This discovery led over time to the development of other GLP-1 analogs with more stable degradation properties, including liraglutide (half-life 11–13 h) and semaglutide (half-life 144–168 h).

Initial drug development focused on GLP-1 receptor agonists (GLP-1 RAs) as an innovative treatment for type 2 diabetes mellitus through blood glucose reduction. Trials in adults with diabetes uncovered unexpected benefits beyond blood glucose regulation, including appetite reduction and prolonged satiety. Soon investigators began studying GLP-1 RAs as a weight loss treatment in obesity, and landmark clinical trials demonstrated weight loss at levels beyond any previously studied weight loss medications. Benefits were discovered to extend to other conditions, including hypertension, hyperlipidemia, sleep apnea, kidney disease, metabolic dysfunction-associated steatohepatitis, and cardiovascular disease prevention. However, durable weight loss with GLP-1 RAs requires continued treatment and rebound weight gain is common after drug discontinuation [23].

The most common adverse effects are dose-dependent and gastrointestinal in nature, including diarrhea, constipation, nausea, and vomiting. These are typically self-limiting or resolve with dose reduction, but can lead to drug discontinuation in some patients. Rapid weight loss can lead to hair loss and muscle loss. More serious gastrointestinal adverse effects include pancreatitis, gastroparesis, bowel obstruction and biliary disease [24, 25]. Mean resting heart rate can be slightly elevated in certain populations with GLP-1 RAs, but the cardiac benefits include reduced incidence of death from cardiovascular causes, nonfatal myocardial infarction, or nonfatal stroke (hazard ratio (HR) 0.80; 95% CI, 0.72–0.90, p < 0.001) [26]. Animal studies and early human studies had conflicting findings about the risk for thyroid cancer, but more recent studies suggest no increased risk of developing thyroid cancer and postulate that previously reported higher rates of thyroid cancer may have been due to surveillance bias rather than a direct oncogenic effect [27, 28]. Most GLP-1 RAs remain contraindicated in patients with a personal or family history of medullary thyroid carcinoma or in those with multiple endocrine neoplasia syndrome type 2 (MEN 2). When used in adults, GLP-1 RAs do not require dose adjustment for kidney impairment, although data is limited on its use with kidney failure. In children with kidney disease, GLP-1 RAs remain unstudied and would represent an off-label treatment for children with kidney impairment.

##### Semaglutide

Semaglutide is a GLP-1 RA with a once-weekly dosing regimen that was approved by the FDA in 2022 as adjunct weight loss therapy for children with obesity ≥ 12 years (BMI ≥ 95th percentile). In the STEP TEENS trial, a 68-week double-blind study comparing semaglutide to placebo in adolescents with obesity and at least one weight-related coexisting condition, semaglutide treatment decreased BMI by 16.7% relative to the placebo, and 76% of the semaglutide group had ≥ 5% reduction in BMI, compared to 23% in the placebo group [29]. Very large reductions in body weight were noted in the semaglutide group, with 37% having a ≥ 20% loss of body weight compared to 3% of the placebo group. Other cardiometabolic risk factors, including waist circumference, glycated hemoglobin levels, and certain lipid levels were improved with semaglutide compared to placebo. Gastrointestinal adverse events were reported in both groups, 62% with semaglutide compared to 42% with placebo.

##### Liraglutide

Liraglutide, a once-daily injected GLP-1 RA was approved in 2010 for adults with type 2 diabetes mellitus as an adjunct to diet and exercise. The indication was expanded to chronic weight management for adults with obesity or overweight and with at least one weight-related comorbidity in 2014, followed by an indication for reduction in major adverse cardiovascular events in adults with type 2 diabetes and cardiovascular disease. The pediatric indications were approved in 2019, first for children ages 10 and older with type 2 diabetes, and in 2020 for adolescents 12 and older with obesity.

A limited number of randomized trials have been conducted to assess the weight loss effect of liraglutide in children without diabetes. A 2023 systematic review [30] identified only 3 trials, two of which were small (n = 21–24) and of short duration (1–8 weeks of treatment) [31, 32]. The largest trial included 251 pubertal adolescents (12 to < 18 years of age) with obesity and tested liraglutide 3.0 mg against placebo for 56 weeks of treatment [33]. This study observed a ≥ 5% reduction in BMI in 43.3% of the liraglutide group, compared to 18.7% of the placebo group; and a reduction of ≥ 10% in BMI was observed in 26.1% of the liraglutide group, compared to 8.1% of the placebo group. These trials showed that liraglutide was generally well tolerated in adolescents and children, with a side effect profile similar to those seen in adult studies and other medications within the same GLP-1 RA class.

##### Future directions with GLP-1 RAs

In terms of short-term weight loss, liraglutide and semaglutide are the most effective pharmacologic agents currently available in the United States for pediatric obesity. The number of adolescents and young adults who were prescribed GLP-1RAs increased 600% in recent years [34]. While head-to-head trials have not been conducted in children, in non-diabetic adults with obesity and overweight the STEP-8 trial showed that semaglutide produces significantly greater weight loss than liraglutide [35]. Dual agonists that target both GLP-1 and glucose-dependent insulinotropic peptide (GIP) are now widely used in adults and have been shown to produce even greater weight loss than semaglutide [36]. One dual agonist, tirzepatide, is currently being studied in adolescents [37], while triple agonists targeting GLP-1, GIP, and glucagon are being studied in adults and hold promise to further revolutionize obesity pharmacotherapy [38]. All of the previously discussed GLP-1 RAs are injectable medications, but in December 2025 an oral once-daily pill (oral semaglutide) became the first oral GLP-1RA approved for chronic weight management in adults with obesity and overweight. Studies are needed to investigate their use in children.

GLP-1 RAs are a highly effective short-term treatment for pediatric obesity, but additional long-term data will be needed to identify the potential for harms from long-term use and how to attenuate rebound weight gain after treatment discontinuation. Continued research will be important to better understand the impact on cardiometabolic outcomes in pediatric populations over time.

Given the worldwide prevalence of obesity, which disproportionately affects marginalized populations, it is critical to ensure that trials of newer therapies include diverse populations. A recent meta-analysis reported that participants who self-identified as White comprised 79% of those enrolled in GLP-1 trials, representing a substantial overrepresentation that was especially pronounced in studies conducted in Brazil and South Africa [39]. In addition, 84% of trial sites were located in high-income countries, likely reflecting broader structural disparities in global health research capacity.

High drug costs combined with restricted insurance coverage for GLP-1 RAs can lead to high out-of-pocket-expenses for many patients. For lower income patients, this may present an insurmountable access barrier. Like most weight management strategies, GLP-1 RAs may require years of continuous use to maintain weight loss, and it remains unclear whether the high upfront drug costs will justify the long-term savings from reduced future complications of obesity.

#### Phentermine + topiramate combination therapy

The once-daily combination product phentermine/topiramate was approved by the FDA in 2022 for weight management in adolescents with obesity ≥ 12 years old. This medication provides appetite suppression via two pathways, combining the sympathomimetic action of phentermine (described below) with the increased satiety associated with topiramate. While the exact mechanism of increased satiety with topiramate is unknown, it is hypothesized to occur via activation of GABA-A receptors, affecting 1) the leptin pathway in the lateral hypothalamus, and 2) the transmission of taste for carbonation and sweet taste at the taste bud level [40]. Topiramate may also reduce food cravings. It is postulated that the combination product is more effective for weight loss than either monotherapy, possibly due to the immediate release effect of phentermine earlier in the day that is supplemented by the extended-release action of topiramate to maintain appetite suppression into the evening hours [41].

Several high-quality large randomized trials in adults have shown that phentermine/topiramate results in effective weight loss, with mean difference of percentage body weight change –7.98 (95% CI –9.27 to –6.69) [42]. In adolescents 12–17 years old with obesity, a 56-week double-blind clinical trial in 2022 showed a BMI reduction of –10.4% with the highest dose of phentermine/topiramate as an adjunct to lifestyle therapy, and 68% of those in the highest-dose group had ≥ 5% BMI reduction, versus 29% in the placebo group [43]. Improvements were also noted in waist circumference and levels of triglycerides and high-density lipoprotein (HDL) cholesterol.

Milder adverse effects include headaches, paresthesia, dry mouth, dizziness, insomnia, and palpitations. Serious side effects can include acute angle-closure glaucoma, suicidal ideation or behavior, and increased heart rate [44, 45]. The known teratogenic effects of topiramate should be considered in patients with childbearing potential, and use of phentermine/topiramate requires a Risk Evaluation and Mitigation Strategy (REMS) to prevent prenatal exposure. Due to the risk of topiramate-induced renal tubular acidosis (RTA), children with a history of nephrolithiasis or known RTA should not receive phentermine/topiramate. Phentermine/topiramate is a valuable pharmacologic option for the treatment of adolescent obesity when used with appropriate clinical monitoring.

#### Orlistat

Orlistat is a gastrointestinal lipase inhibitor that promotes weight loss by interfering with dietary fat absorption in the gastrointestinal tract. This drug is preferred by some because its mechanism of action is peripheral and has no central nervous system activity. Orlistat was FDA-approved in 2003 as an anti-obesity medication for adolescents ≥ 12 years old and is available at lower doses without a prescription. The largest randomized trial of orlistat in adolescents (n = 539) was a multicenter, 54-week placebo-controlled trial which found that 26.5% of the orlistat group had ≥ 5% reduction in BMI compared with 15.7% of the placebo group (p = 0.005); and 13.3% of the orlistat group had ≥ 10% reduction in BMI compared with 4.5% of the placebo group (p = 0.002) [46]. However, adherence rates may be compromised by intolerable gastrointestinal side effects, including steatorrhea, flatulence, and fecal urgency.

In addition, observational studies have suggested an association between orlistat-induced hyperoxaluria and acute kidney injury and chronic kidney disease (CKD) [47, 48]. The proposed mechanism mirrors enteric hyperoxaluria, in which unabsorbed dietary fat binds enteric calcium, making less calcium available to bind and sequester oxalate in the gut, leading to oxalate nephropathy and acute kidney injury. Due to the less favorable side effect profile, orlistat is rarely used as a first-line agent. It may be considered in specific situations where centrally acting agents are contraindicated or less desirable. Its role in treating obesity in adolescents has now largely been displaced by newer agents with greater efficacy and fewer side effects.

#### Phentermine monotherapy

Phentermine is an indirect-acting sympathomimetic amine that suppresses the appetite by increasing synaptic norepinephrine release in the hypothalamus, reducing the perception of hunger and increasing energy expenditure [41]. With a chemical structure similar to amphetamines, phentermine is classified as a schedule IV controlled substance because its stimulant effects have a theoretical potential for abuse and dependence. In 1959 phentermine was approved for short-term use (12 weeks or less) in adults and older teens (≥ 16 years old). While it remains unapproved for children younger than 16 years old, in practice it has been used off-label for children 12 and older with parental consent, and is often used long-term despite the short-term labelling. Treatment discontinuation after long-term use requires very slow tapering (across 1 to 2 years) to avoid rebound weight gain.

The data to support the use of phentermine monotherapy in children is strikingly lacking. A review published in 2023 identified only three studies in children from 1966 to 2022, each of which reported the weight loss outcome differently [41]. Although two single center case series suggested benefit, the only placebo controlled randomized trial in children (n = 68 and published in 1966) showed clinically insignificant weight loss at 12 weeks compared to placebo (average weight loss 1.4 kg versus 0 kg respectively, p values not reported) [49]. Side effects of phentermine can include palpitations, insomnia, constipation, xerostomia, agitation, increased blood pressure (BP) or heart rate, and anxiety. Given the limited efficacy data, side effect profile and potential for misuse, phentermine monotherapy is not considered a first line agent for pediatric obesity. However, it may be considered as an adjunctive treatment in older adolescents (≥ 16 years) with severe obesity who have not responded adequately to behavioral interventions or who cannot access or tolerate other pharmacologic agents.

### Off-label weight loss pharmacotherapy

#### Metformin

Metformin, a widely used treatment for type 2 diabetes mellitus, improves glycemic control by increasing peripheral insulin sensitivity and decreasing hepatic gluconeogenesis. Additional sites of action on the gastrointestinal tract have been described, including inhibition of intestinal glucose absorption, alteration of gut microbiome, and increased intestinal secretion of GLP-1 [50]. The initial studies suggesting a weight-loss benefit showed either weight-neutral or weight-loss effects compared to other diabetes medications in adults, including sulfonylureas and glitazones [51–53]. The largest study to show weight loss with metformin was the Diabetes Prevention Program, which assessed the impacts of metformin in over 3000 adults at high risk for type 2 diabetes mellitus and observed an average 2.5 kg weight loss among participants randomized to metformin, an effect that persisted up to 10 years with high adherence to metformin [54].

A systematic review and meta-analysis published in 2021 identified twenty-four randomized trials assessing the efficacy of metformin for pediatric obesity [55]. The trials were of varying quality and showed modest decreases in BMI (range of mean values: − 2.70 to 1.30 kg/m^2^ vs, − 1.12 to 1.90) and a relatively high frequency of gastrointestinal side effects, including diarrhea, nausea, and abdominal pain. The study also found inconclusive evidence on the risk for liver toxicity with metformin when used for obesity.

Overall, the available evidence for metformin therapy as an adjunct to lifestyle interventions suggests a modest benefit at best in children. However, given its availability and low-cost, it continues to be used off-label for children who have limited access to other pharmacologic options.

#### Combination naltrexone/bupropion

Bupropion is a norepinephrine/dopamine reuptake inhibitor that was approved in the 1990s for depression treatment and smoking cessation in adults. Subsequent studies on its use for treatment of depression had conflicting reports on its effects on weight. A systematic review of studies in adults suggested that overall, it was more likely to be associated with weight loss than weight gain [56]. The mechanism of action for weight loss is likely related to the pro-opiomelanocortin (POMC) pathway in the hypothalamus. POMC neurons release α–melanocyte-stimulating hormone (MSH) and β-endorphin. α–MSH has an anorectic effect, and β-endorphin provides autoinhibitory feedback, inactivating the anorectic effect of α–MSH. Bupropion stimulates the POMC neurons, resulting in increased anorexic effect through α–MSH release [57]. Studies in children have identified small and unsustained reductions in BMI when bupropion monotherapy is used for weight loss [58, 59].

An extended-release, fixed-dose combination product containing bupropion and naltrexone, an opioid antagonist, was approved in 2014 for adults with obesity. The naltrexone component blocks the autoinhibitory feedback of β-endorphin in the POMC pathway. Naltrexone alone cannot produce weight loss, but when combined with bupropion it synergistically enhances the effect of bupropion on POMC signaling [60]. In adults, a meta-analysis of 6 randomized trials in nearly 10,000 adults found that naltrexone/bupropion and intensive behavioral therapy produced a 9.3% weight loss at 12 months, compared to 5.1% with placebo [42]. Naltrexone/bupropion is contraindicated in uncontrolled hypertension, seizure disorder (current, history of, or risk for), chronic opioid or opiate agonist use, acute opiate withdrawal, and concurrent use of monoamine oxidase inhibitors. Most importantly, naltrexone/bupropion carries a boxed warning for the risk of suicidal thoughts and behavior in children, adolescents, and young adults (< 24 years old). If used off-label for weight loss in pediatric populations, it must be avoided in individuals with depression or anxiety.

#### Topiramate monotherapy

Topiramate is an effective and widely used treatment for migraines and seizure prophylaxis [61]. Adolescents and adults with and without obesity who are prescribed topiramate monotherapy for seizure control or migraine prophylaxis have been observed to experience appetite suppression and weight loss [62–65]. The hypothesized mechanism for appetite suppression with topiramate, via GABA-A receptor blockade, is described in the phentermine/topiramate section above. Very few studies have specifically examined topiramate monotherapy as a weight loss treatment for pediatric obesity. An eight-week placebo-controlled randomized trial among adolescents and adults with Prader Willi syndrome (participant mean age 24 years) found no significant reductions in BMI, but did observe improvements in binge eating behavior [66]. A pilot placebo-controlled randomized trial in adolescents with severe obesity (n = 21) found improvements in visceral fat and cholesterol level, but without significant BMI reduction [67]. Side effects include fatigue, dizziness, cognitive slowing, numbness of extremities, nephrolithiasis, glaucoma, metabolic acidosis, and leukopenia. The quality of evidence to support the use of topiramate monotherapy for weight loss in children remains lacking and it is unlikely to be studied further given the advent of other classes of highly efficacious weight-loss treatments. Among children 12 years and older who require migraine prophylaxis, topiramate may be a preferred option for those individuals who also have obesity.

## Obesity medications and hypertension

Obesity is a well-recognized risk factor for the development of childhood hypertension. A large retrospective cohort study including over 100,000 subjects across 3 health systems in the United States observed a more than threefold risk of incident hypertension among children who became obese or maintained obesity over three year follow-up compared to those who maintained a healthy weight [68]. The pathogenesis of obesity-induced hypertension is multifactorial, involving increased activity of the sympathetic nervous system (SNS), oxidative stress-induced inflammation, activation of the renin–angiotensin–aldosterone system (RAAS), and compression of kidneys due to fat accumulation [69]. The causative role of SNS overactivity is illustrated in studies that show that Native American Pima Indians, who have lower basal muscle SNS activity than White individuals, experience lower rates of hypertension despite very high rates of obesity [70]. Disruption of normal RAAS suppression and higher SNS activity interact to stimulate renin secretion, which increases BP.

The management of obesity-related hypertension should address the reduction of BMI in addition to treatments aimed at reducing BP. A prospective cohort study from a Swedish national registry among children receiving behavioral lifestyle modifications evaluated the effect of weight reduction on BP [71]. The study found that both systolic and diastolic BP decreased when lower BMI was achieved over 32-month follow-up, and obesity treatment failure significantly increased the risk of developing hypertensive BP; HR 1.81 (99% CL 1.38–2.37). Importantly, it was noted that behavioral treatment failed to lower BP in 27% of children with hypertension at the time of treatment initiation, highlighting the role of antihypertensive medication in these patients.

While antihypertensive medications remain the cornerstone of pharmacologic treatment of hypertension, the advent of highly effective anti-obesity medications has opened the possibility that these medications may also have important BP reduction effects. The STEP-1 trial, which enrolled nearly 2000 adult participants with overweight and obesity but without diabetes, and found that after 68 weeks of treatment the semaglutide group had a greater systolic BP reduction (mean difference –5.10 mmHg; 95% CI –6.34 to –3.87) compared to placebo [72]. Moreover, the STEP-1 extension trial found that one year after withdrawal of treatment, all participants experienced a reversion of mean systolic BP and diastolic BP back to baseline levels in addition to regaining two-thirds of the weight previously lost [23]. Furthermore, the SURMOUNT-1 ambulatory BP monitoring substudy assessed the BP lowering effect of tirzepatide, a dual-action GIP (glucose-dependent insulinotropic polypeptide) and GLP-1 RA [73]. After 9 months, up to an 11 mmHg reduction in 24-h systolic BP was noted in the tirzepatide group compared to placebo. A 2025 meta-analysis of 33 randomized trials assessed the magnitude of BP reduction with GLP-1 RA combined with lifestyle modification versus control groups with placebo and lifestyle modification, and found greater reductions; systolic BP (mean difference: − 3.99 mmHg, 95% CI: − 5.66, − 2.33, *P* < 0.001), diastolic BP (MD: − 1.11 mmHg, 95% CI: − 1.71, − 0.42, *P* = 0.002) [74].

A recent systematic review and meta-analysis of the efficacy and safety of GLP-1 RAs in children and adolescents with obesity or type 2 diabetes included randomized trials published through February 2025 [75]. The nine trials that reported BP outcomes among children with obesity (total n = 773) showed a significant overall reduction in systolic BP in the GLP-1 RA group (mean difference –2.85 mmHg; 95% CI –4.32 to –1.38) compared to placebo, over a mean 4 month follow-up. Several post-hoc analyses of placebo-controlled trials of GLP-1 RAs have observed that the BP lowering effect may not be completely explained by reductions in body weight [76]. The postulated mechanisms suggested by human and animal studies include augmentation of natriuresis, amelioration of RAAS activity, and direct vasodilation through activation of GLP-1 receptors in endothelial cells and vascular smooth muscle cells [76]. Longer term studies that evaluate cardiovascular morbidity and mortality are needed in children to better understand the potential for BP lowering effects of anti-obesity medications in this population.

## Obesity medications and chronic kidney disease

The bidirectional relationship between CKD and obesity in children represents a growing challenge in clinical management, with each condition exacerbating the progression and complexity of the other. Obesity is an established independent risk factor for CKD onset and progression, mediated by multiple interacting mechanisms, including insulin resistance, hypertension, chronic low-grade systemic inflammation, oxidative stress, lipotoxicity, and RAAS-mediated hyperfiltration [77, 78]. The complex interplay of these factors contributes to the development of obesity-related glomerulopathy, a distinct pathological entity characterized by glomerulomegaly and oligonephronia. Over time, these lead to progressive glomerulosclerosis and decline of kidney function. RAAS upregulation induces hyperfiltration, which increases the risk of hypertension and proteinuria. Excess fat deposition in the kidneys, progressive lipotoxicity, has a nephrotoxic effect induced by oxidative stress and fuels ongoing inflammation, leading to podocyte damage, glomerulosclerosis, and renal fibrosis. Moreover, insulin resistance impairs insulin receptor function at the podocyte level, which weakens the integrity of the podocyte barrier and alters podocyte adaptive functions [79].

The Pediatric Renal Nutrition Taskforce, an international group of pediatric nephrologists and dietitians, developed clinical practice recommendations in 2022 on the management of obesity and metabolic syndrome in children with CKD [80]. Their recommendations generally prioritize non-pharmacologic management, focusing on comprehensive lifestyle interventions including nutritional counseling, physical activity, and behavioral modifications. Given the paucity of randomized trials of anti-obesity medications among children with kidney disease, the Taskforce recommended against the use of anti-obesity medications in children with CKD stages 2 through 5D or with a kidney transplant.

Currently, there are no ongoing clinical trials investigating GLP-1 RA medications in children with CKD, but landmark studies have shown kidney benefits in adults with obesity, with and without diabetes [81, 82]. Initial studies of GLP-1 RAs in adults with diabetes noted in secondary and exploratory analyses that albuminuria was consistently reduced or prevented with GLP-1 RA treatment [83, 84]. The FLOW trial was the first large GLP-1 RA trial to study major kidney disease events as the primary outcome among adults with type 2 diabetes and CKD, with a composite outcome of onset of kidney failure, a 50% or greater reduction in estimated GFR from baseline, or death from kidney-related or cardiovascular causes [81]. Among the 3533 randomized participants, the semaglutide group had a 24% lower risk for the primary outcome over 3.4-year follow-up than the placebo group (HR 0.76, 95% CI 0.66 to 0.88; p = 0.0003). A pre-specified secondary analysis of the SELECT trial, which included adults with overweight/obesity and established cardiovascular disease but without diabetes, showed similar long-term kidney benefits in the semaglutide group, who had a 22% lower risk for the composite kidney endpoint of death from kidney disease, initiation of chronic kidney replacement therapy, onset of persistent eGFR < 15 ml/min/1.73 m^2^, persistent ≥ 50% reduction in eGFR, or onset of macroalbuminuria (HR 0.78, 95% CI 0.63 to 0.96, p = 0.02) [82].

Improvements in kidney disease risk factors, including hypertension, obesity, hyperglycemia, and cardiac disease may explain some, but not all of the renoprotective effects of GLP-1 RA treatment. Several human physiology studies and preclinical studies suggest additional direct kidney effects, including reduction in proinflammatory cytokines and chemokines and reduction in intra-kidney fat accumulation [85]. In 2025, semaglutide became the first GLP-1 RA to be approved by the FDA for a CKD indication, to reduce the risk of worsening kidney disease, kidney failure, and cardiovascular death in adults with type 2 diabetes and CKD.

Since 2020, the KDIGO guidelines have recommended the use of a long-acting GLP-1 RA for adults with type 2 diabetes and CKD who have not achieved individualized glycemic targets despite use of metformin and SGLT2 (sodium-glucose cotransporter 2) inhibitor treatment (Level 1B) [86]. An additional practice point recommends prioritization of GLP-1 RA agents with documented cardiovascular benefits.

## Surgical options

Metabolic and bariatric surgery is an effective treatment for adolescents with class 2 or 3 obesity and is generally considered when lifestyle and pharmacologic interventions have failed. The most common procedures in adolescents are Roux-en-Y gastric bypass or vertical sleeve gastrectomy. The Teen-Longitudinal Assessment of Bariatric Surgery (Teen-LABS) study showed substantial and durable benefits, including 27% weight loss at 3 years and sustained improvements in diabetes, hypertension, and dyslipidemia, although surgery carries risks and requires specialized multidisciplinary care and lifelong follow-up [87].

## Conclusion

Lifestyle and behavioral modifications instituted through partnership between the patient, family, and a multidisciplinary medical team remain the first-line treatment for pediatric obesity. However, as newer pharmacologic treatments for obesity become commonplace for adults, children with obesity may soon have many more therapeutic options.

Of the currently available pharmacologic options in pediatrics, GLP-1 RAs (including formulations with GIP and glucagon receptor agonists) are the most promising weight loss medications. Combination phentermine/topiramate is also an effective and well tolerated pharmacologic option with FDA approval in adolescents. Older medication options such as orlistat and phentermine monotherapy demonstrate modest reductions in weight, but use has been limited due to general tolerability and safety concerns. Off label medications including topiramate monotherapy, metformin, and naltrexone/bupropion may be considered, but overall lack sufficient long-term evidence of efficacy and remain unapproved for weight loss in children.

As the burden of childhood obesity continues to grow, continued research into anti-obesity management is critical. Several unanswered questions around the safety and costs of long-term use of weight loss pharmacotherapy must be addressed before widespread uptake is possible. Currently approved medications should be studied for long-term outcomes with extended treatment times, and outcomes other than BMI such as hypertension, kidney function, insulin sensitivity, or other cardiometabolic outcomes must be included. The natural history of obesity across the lifespan is marked by periods of treatment response followed by relapse when treatment is discontinued. Therefore, sustained, coordinated care is necessary to support obesity management from childhood and adolescence into young adulthood.

## Supplementary Information

Below is the link to the electronic supplementary material.

Graphical abstract (PPTX 107 KB)

## Data Availability

Data sharing not applicable to this article as no datasets were generated or analyzed during the current study.
